# Association between TNF-α rs1799964 and RAF1 rs1051208 MicroRNA binding site SNP and gastric cancer susceptibility in an Iranian population 

**Published:** 2017

**Authors:** Shaghayegh Baradaran Ghavami, Vahid Chaleshi, Shaghayegh Derakhshani, Pedram Aimzadeh, Hamid Asadzadeh-Aghdaie, Mohammad Reza Zali

**Affiliations:** 1 *Basic and Molecular Epidemiology of Gastrointestinal Disorders Research Center, Research Institute for Gastroenterology and Liver Diseases Shahid Beheshti University Medical Sciences, Tehran, Iran*; 2 *Gastroenterology and Liver Diseases Research Center, Research Institute for Gastroenterology and Liver Diseases, Shahid Beheshti University of Medical Sciences, Tehran, Iran*

**Keywords:** single nucleoid polymorphism, Gastric cancer, Tumor necrosis factor, microRNA

## Abstract

**Aim:**

The aim of this study was to find the relationship between rs1799964 in TNF-α gene as well as rs1051208 of RAF1 gene SNPs on GC in an Iranian population.

**Background:**

Gastric cancer (GC) is the second leading cause of cancer-related death worldwide after lung cancer. Tumor necrosis factor (TNF) is one of the most important factors in the pathogenesis of this cancer. Single nucleotide polymorphisms have a principle role in gene expression of TNF-α and miRNAs which may lead to gastric cancer.

**Methods:**

In a case-control study, we investigated the risk of GC in 198 Iranians. For this purpose, 5 mL of peripheral blood was collected in EDTA –containing tube and genomic DNA was isolated. Genotyping of SNPs was also performed by PCR-RFLP; to approve the outcome, 10% of genotyping results with RFLP were sequenced.

**Results:**

The comparison between case and control groups revealed a significant association between the rs1051208 C allele of RAF1 gene and GC (P = 0.04). We did not observe any remarkable association between TNF-α -1031 in gastric cancer patients and the healthy control group.

**Conclusion:**

The results indicated that C allele in RAF1 gene plays a role in susceptibility to gastric cancer. Therefore, SNPs are among notable biomarkers for predicting susceptibility to dreadful diseases, especially cancers.

## Introduction

 Cancers constitute a major public health concern, especially gastric cancer (GC) which is the second leading cause of death among cancers (1, 2). The incidence of gastric cancer varies in different geographical regions. It is more common in East Asia, East Europe and part of Latin America (3-5). In Iran, people of the northern and north western areas are at high risk for gastric cancer (6, 7). The disease is more common in male gender both in Iran and other parts of the world (4, 7, 8). H. pylori infection is a considerable (but not the sole) risk factor for developing the disease (8-10). This cancer is a multi-factorial disease, and factors including diet (high salt, drinking alcohol, insufficient level of antioxidants), smoking, genetic variants and environmental agents are considered to affect its prevalence (4, 11).

Genetic factors, including expression or silence of some genes, determine an individual’s susceptibility to cancer. Chronic inflammatory response induced by H. pylori infection (12) has been reported to be one of the early phases in the development of gastric cancer (13). Inflammation occurs as a result of cytokine secretion, and the most important pro-inflammation cytokines are IL-8, IL-6, IL1β, TNF-α, and IFN-γ (5, 10, 14). Among them, tumor necrosis factor (TNF) is one of the most important factors in development of GC which can also inhibit gastric acid secretion (5, 10, 14, 15). TNF-α expression significantly increases in the sera of the patients with advanced gastric cancer (16, 17). Increasing TNF-α has also been detected in men with H. pylori infection (1, 18, 19). TNF-α is secreted by macrophages, monocytes, T cell, NK-cells; it is located on the class III region of histocompatibility complex on chromosome 6 (1). The expression of TNF-α is controlled by single nucleotide polymorphisms (SNPs). To date, different SNPs have been identified on TNF-α promoter, some of the most important of which include -308, -238, -1031, and -875 (1, 13). TNF-α -1031 polymorphism has been investigated in several cancers, autoimmune diseases, hepatitis, and infectious diseases (20). In all the above mentioned diseases, high levels of TNF-α cytokine have been reported (21). TNF-α -1031 polymorphism has been reported to affect the risk of gastric cancer (20). 

Recent studies indicate that increased or decreased expression of some genes has an important role in cancer development. In spite of genetic variation, gene regulator crucially affects this potency, as well. One of the important agents in regulation of gene expression is a small, noncoding-protein RNA molecule, called microRNAs (miRNAs). MicroRNAs are single-stranded molecules of about 18-24 nucleotides, which are highly conserved and function post-transcriptionally. This means that they can directly interact with the 3΄ untranslated regions (3΄UTRs) of messenger RNAs (22). The binding of microRNAs inhibits its expression to messenger RNAs; single-nucleotide polymorphisms can affect this process and thus increase the risk of different diseases, especially cancer (23-25). Polymorphism of miRNAs has an impact on alternation of cancer-related genes, such as RAF-1, that could determine probable susceptibility to cancer (26). RAF-1 has been found to be associated with different cancers, especially colorectal cancer (CRC) (24-26). To date, SNPs of the gene RAF-1 (rs1051208) have not been evaluated in gastric cancer. 

We investigated two single nucleotide polymorphisms (SNPs) of TNF-α -1031 and RAF-1 rs1051208- in a gastric cancer in an Iranian population. 

## Methods

**Study population and data collection**

This is a case-control study on 99 patients with gastric cancer and 99 healthy controls referred to Taleghani Hospital, Iran, Tehran. Those with positive endoscopic and pathologic findings for gastric cancer were included. Controls were checked for inflammatory diseases and malignancy. The study was approved by the ethics committee of the Research Institute for Gastroenterology and Liver Diseases, Shahid Beheshti university of Medical sciences, Tehran, Iran. 

**Genotyping**

Initially, 5 mL of peripheral blood was collected in EDTA-containing tube and genomic DNA was isolated by salting method extraction (27). Genotyping was performed based on polymerase chain reaction –restriction fragment length polymorphism (PCR-RFLP). Also, the TNF-α (-1031) polymorphism was detected using PCR-RFLP method. The 186-base pair PCR product was generated in 25 μL final mix containing 2.5 μL of 10X PCR buffer, 100 ng DNA template, 0.5 pM of each primer Table1, 0.75 mM MgCl2 and 0.5 μM of each dNTP and 1.5 unit of Taq DNA polymerase. The amplification protocol was carried out under the following conditions: a denaturation step for 4 min at 950C followed by 37 cycles of a denaturation at 940C or 30 s, annealing at 62.50C for 30 s, extension at 720C for 40 s. A final extension was performed for 10 min at 720C. DNA bands were detected using ethidium bromide staining after being run on 1% agarose gel (Roche, Germany). The PCR products were digested with 3 unit BbsI (Fermentase) endonuclease overnight at 370C (Table 1). The digested PCR products were analyzed using electrophoresis on 2.5% agarose gel (Roche, Germany) and staining by ethidium bromide. The result of digestion is TT 186bp, TA 186bp, 106bp, 80bb, AA 106bp, 80bp.2

 Also, RAF-1 SNP was detected with 669 bp PCR product in 25 μL final cocktail. 100 ng genomic DNA was added to 2.5 μL of 10X PCR buffer (10 mM Trich-choloride, 50 mM chloride potassium 0.1%, Tritiun X-100) (genefanavaran-Iran), 1 μL MgCl2(genefanavaran-Iran), 0.5 μL dNTP (genefanavaran-Iran), 0.4 pM of each primer (Table1), 2 units of Taq DNA polymerase (genefanavaran-Iran) under the following conditions: a denaturation step for 5minutes at 940C followed by 37 cycles of a denaturation for 45 seconds at 940 C, annealing for 30 seconds at 560 C , extension for 45 seconds at 720C and a final extension for 10 minutes at 720C in a thermocycler (Eppendorf, Germany). DNA bands were detected using ethidium bromide staining after being run on 1% agarose gel (Roche, Germany). The PCR products were digested with 4 units of NsbI (Fermentase) endonuclease overnight at 370C. The digested PCR products were analyzed using electrophoresis on 2.5% agarose gel (Roche, Germany) (Table 1) and staining by ethidium bromide. The digested fragments are TT 669 base pair, TC 669bp, 389bp, 280bp, CC 389bp, 280bp.

**Statistical Analysis**

Statistical analysis was performed using the Statistical Package for Social Sciences software (SPSS) version 11.0. Logistic regression analysis was applied to estimate odds ratios (OR) and 95% confidence intervals (CI). The Hardy-Weinberg cytokine gene polymorphisms equilibrium was examined by chi-square test. Age, sex, and recruitment source (two subject groups) were adjusted to exclude any potential confounding. 

## Results

Totally, 198 GC cases and controls (99 in each group) were genotyped. More specifically, the case group consisted of 46.5% males and 53.5% females while the control group included 66.7% males and 33.3% females (Table 2). Patients in the case and control groups were significantly different regarding their age and gender (p<0.05); however, smoking habits and BMI were similar between them (p>0.05).

Comparing the cases and controls revealed a statistically significant difference in the distribution of RAF1 gene rs1051208 genotypes between them (P < 0.001).We also found a significant association between the rs1051208 C allele of RAF1 gene and higher risk of GC (adjusted OR = 0.422, 95% CI: 0.18-0.967, P = 0.04). According to statistical analysis performed, no significant difference was found in the rs1799964 SNP of TNF-α gene between the two groups (P> 0.05; Table3).

## Discussion

Evidence supports the fact that TNF induces a ceramide activated protein kinase (CAPK) which can directly phosphorylate and active RAF-1(28, 29). RAF-1 was reported to have an oncogenic function due to its regulatory role as the main mediator in mitochondrial apoptotic pathway (30, 31). Different studies have shown that activation of RAF-1 is strongly associated with resistance to apoptosis which might be the major cause of uncontrolled cell cycle divisions in cancer (30). These two SNPs on TNF and RAF-1gene are important because of their effect in regulating expression proteins, leading to inflammation.

**Table 1:** Primers and restriction enzymes

SNP	Primer	Reign	Product size	Restriction Enzyme	fragment
**TNF-α rs1799964**					
Forward	5΄-CTTCAGGGATATGTGATGGACTC-3΄	Promoter	186	BbsI	C:186 T:106+80
Reverse	5΄- ACATCTCCCCAGAGGTCTCC-3΄				
**RAF1 rs1051208 **					
Forward	5΄-CTCCAACACTCTCTACCGAAGATC -3	3΄UTR	669	NsbI	C: 669 T:389+280
Reverse	5΄-AAGAAAAGTTCCATAGTACCAAAG-3΄				

**Table 2:** Characteristics of study participants including GC cases and healthy controls

	Controls	Patients
Age	46.64±16.9	60.27±13.4
Gender		
male	66(66.7%)	46(46.5%)
female	33(33.3%)	53(53.5%)
BMI	25.21±3.9	25.23±4.5
Smoking		
Yes	6(00%)	16(16.2%)
No	93(00%)	83(83.8%)

**Table 3:** Genotypes and allele frequencies of TNF-α and RAF-1 gene polymorphism in gastric cancer and controls group

SNP	Healthy control (n=99) n(%)	Gastric cancer (n=99) n(%)	OR(95%CI)	P value
TNF-α rs1799964				
TT	75 (75.8)	64 (64.6)	1.00(Reference)	
TC	20 (20.2)	29 (29.3)	0.258(0.73-3.24)	0.25
CC	4 (4.0)	6 (6.1)	1.780(0.433-7.31)	0.42
T allele	170 (85.9)	157(79.3)	1.00(Reference)	
C allele	28(14.1)	41 (20.7)	1.51(0.83-2.03)	0.08
RAF1 rs1051208				
TT	78 (78.8)	91 (91.9)	1.00 (Reference)	
TC	21 (21.2)	6 (6.1)	0.018 (0.007-0.049)	1
CC	-	2 (2.0)	0.037 (0.008-0.178)	0.999
T allele	177(89.4)	188 (94.9)	1.00 (Reference)	
C allele	21 (10.6)	10(5.1)	0.422(0.18-0.967)	0.04

Furthermore, RAF-1 as an important microRNA, attaches to its binding site at 3 ´ UTR gene (a polymorphic region) and regulates gene expression. 

We found a significant association between RAF-1 rs1051208 G/C and risk of gastric cancer (P< 0.05). However, we did not observe a statistically significant association between TNF-1031 T/C rs1799964 and cancer (P=0.23). Our result on TNF-1031 polymorphism was similar to those reported in a Korean population (P=0.23) (1). Most of the previous studies on Asian populations have demonstrated findings similar to ours, especially in duodenal ulcer (DC), considering the fact that DC can increase the risk of gastric cancer (1, 31). There is a low acid secretion capacity in gastric cancer whereas duodenal ulcer patients have high levels of gastric acid secretion. Another study on an Italian population also showed no association between this SNP and DC P=0.77 (1, 32, 33). Based on the results of Yang et al in Korea, the polymorphism of TNF-1031T/C increased the risk of gastric cancer only among smokers (20).

Sugimoto et al. reported a significant association between TNF-1031 polymorphism and increased risk of gastric cancer in a Japanese population (P=0.01), and showed that the frequency of C allele TNF-1031 was higher in cases compared to the healthy individuals (34). In contrast, we found low frequency of C allele in Iranian gastric cancer cases.

However, the frequency of -1031 C allele in an Indian population has been reported to be higher than other ethnic groups. The minor allele frequency of our result in control group (14.0) is most similar to the Japanese (14.5). Sugimoto et al. reported that -1031C alleles were actually functional and involved in the susceptibility to gastric ulcer and cancer and can induce higher TNF production, especially in East Asian individuals. High levels of TNF-α were found to be defined by -1031C alleles in an in vitro study. Another SNP on TNF-α promotor is polymorphism -308 that was previously investigated by Seilanian Toosi et al. in an Iranian population and associated with the risk of gastric carcinoma (35).

In a meta-analysis by Loh and colleagues in Singapore, the authors evaluated the TNF-1031 polymorphism in Asians (36). Their results showed no statistically significant association between DU and -1031 T/C polymorphism with OR 0.99 (0.79-1.25). Lack of associations for this polymorphism may reflect genetic heterogeneity in the pathogenesis of gastric cancer (34).

Abedie and colleagues studied TNF-α polymorphism and investigated the seropositivity of anti-H. pylori among Uzbeks. They observed a significant association only for the heterozygotes of TNF-1031 polymorphism and the OR of -1031 TC/CC relative to -1031 TT was not significant. Allele -1031C had a low prevalence for anti H.pylori. Lu and coworkers obtained opposite results (3). Sugimoto suggested a higher susceptibility to gastric ulcer and cancer in East Asian individuals probably through higher TNF-α production (34).

Another SNP, rs1051208 polymorphism on the RAF-1gene, was also investigated in the current study and significantly associated with increased risk of gastric cancer. This is a functional polymorphism because this variation, located in RAF-1mRNA 3 ´UTR, may affect the binding of mir-213 to RAF-1 mRNA (26) due to changes in the mediator function of RAF-1 in apoptosis’s pathway and individual susceptibility to cancer (30). Azimzadeh et al. indicated a statistically significant difference between this SNP and risk of colorectal cancer (CRC) (26). This polymorphism has also been investigated in other cancers, including lung cancer and bladder cancer and even in animal cancers since recent studies suggested microRNA had role in the initiation of cancer (25). More importantly, the increase or decrease of microRNAs is responsible for resistance to chemotherapy (25).

TNF-1031 and RAF-1 have been investigated in few studies, limiting our discussion since we cannot compare our results with other ethnic groups. Also, there are limitations on detailed information of our healthy controls’ and patients’ diet which limit our results.
